# Vascular Smooth Muscle Cell Stiffness and Adhesion to Collagen I Modified by Vasoactive Agonists

**DOI:** 10.1371/journal.pone.0119533

**Published:** 2015-03-06

**Authors:** Zhongkui Hong, Kimberley J. Reeves, Zhe Sun, Zhaohui Li, Nicola J. Brown, Gerald A. Meininger

**Affiliations:** 1 Dalton Cardiovascular Research Center, University of Missouri, Columbia, Missouri, United States of America; 2 Department of Oncology, University of Sheffield, Sheffield, United Kingdom; 3 Department of Pharmacology and Physiology, University of Missouri, Columbia, Missouri, United States of America; LAAS-CNRS, FRANCE

## Abstract

In vascular smooth muscle cells (VSMCs) integrin-mediated adhesion to extracellular matrix (ECM) proteins play important roles in sustaining vascular tone and resistance. The main goal of this study was to determine whether VSMCs adhesion to type I collagen (COL-I) was altered in parallel with the changes in the VSMCs contractile state induced by vasoconstrictors and vasodilators. VSMCs were isolated from rat cremaster skeletal muscle arterioles and maintained in primary culture without passage. Cell adhesion and cell E-modulus were assessed using atomic force microscopy (AFM) by repetitive nano-indentation of the AFM probe on the cell surface at 0.1 Hz sampling frequency and 3200 nm Z-piezo travelling distance (approach and retraction). AFM probes were tipped with a 5 μm diameter microbead functionalized with COL-I (1mg\ml). Results showed that the vasoconstrictor angiotensin II (ANG-II; 10^−6^) significantly increased (*p*<0.05) VSMC E-modulus and adhesion probability to COL-I by approximately 35% and 33%, respectively. In contrast, the vasodilator adenosine (ADO; 10^−4^) significantly decreased (*p*<0.05) VSMC E-modulus and adhesion probability by approximately −33% and −17%, respectively. Similarly, the NO donor (PANOate, 10^−6^ M), a potent vasodilator, also significantly decreased (p<0.05) the VSMC E-modulus and COL-I adhesion probability by −38% and −35%, respectively. These observations support the hypothesis that integrin-mediated VSMC adhesion to the ECM protein COL-I is dynamically regulated in parallel with VSMC contractile activation. These data suggest that the signal transduction pathways modulating VSMC contractile activation and relaxation, in addition to ECM adhesion, interact during regulation of contractile state.

## Introduction

The contractile state of vascular smooth muscle cells (VSMC) determines resistance vessel diameter, which is critical to regulation of tissue blood flow and blood pressure. In addition to the myogenic mechanism, circulating and locally derived factors can induce vasoconstriction or dilatation to modulate vascular tone and hence diameter. As the generation of force by VSMC involves transmission of mechanical force between the cell and the extracellular environment, we hypothesized that changes in the contractile state of VSMC would be accompanied by parallel changes in adhesion to the extracellular matrix (ECM).

Adhesion to the ECM is largely regulated by integrins that are a large family of heterodimeric cell adhesion molecules that anchor cells to ECM and neighboring cells. Additionally, they play key roles in many biological processes including cell motility, cytoskeleton organization, mediating the cell-cell and cell-ECM signals transduction [1–8]. Collagen (COL) is one of the major structural components of ECM within vascular wall and binds to a number of different integrins [9–12]. In this regard, integrin β_1_ is the most predominant integrin expressed on the surface of VSMCs [13] and all COL binding integrins share the common β_1_ subunit. Fibrillar type I collagen (COL-I) is ubiquitously expressed in all vertebrates to provide sufficient mechanical strength for tissues and it is also directly involved in the outside-in signal transduction between the ECM and cells [14]. In addition, integrin activation through inside-out signaling is a well-accepted mechanism for modulation of integrin activation and adhesion with the ECM [15].

Changes in integrin activation and expression have been associated with a number of physiological and pathological processes in the vasculature. This has been proposed to be important for modulation of myogenic phenomena, vascular remodeling, VSMC migration, and VSMC stiffening associated with vascular wall stiffness that occurs in aging and hypertension [16–19]. In previous studies from our laboratory we found that VSMC adhesion to fibronectin (FN) is regulated in parallel with changes in VSMC activation in response to a vasoconstrictor (angiotensin II) or a vasodilator (adenosine) [4]. This raised the question as to whether this also applies to other important vascular wall ECM proteins that interact with VSMCs. Therefore, in this study, we designed experiments to determine whether VSMC adhesion to COL-I is modulated in response to changes in VSMC activation induced by a vasoconstrictor or vasodilator.

## Materials and Methods

### Ethics statement

Male Sprague-Dawley rats were used for this study and were maintained in accordance with the protocol of the *Guide for the Care and Use of Laboratory Animals* (NIH 83-23, revised 1996). The cremaster muscles were excised from anesthetized animals using sodium pentobarbital (Nembutal, 100 mg/kg body weight) given by an intraperitoneal injection. Following sacrifice by anesthetic overdose, the rat was euthanized by thoracotomy. Death will be confirmed by absence of both heartbeat and respiration. All the protocol was approved by the Laboratory Animal Use Committee of the University of Missouri. (Permit number: 7416).

### VSMC isolation and culture

VSMCs were enzymatically isolated from the cremaster muscle first order resistance arteriole using a protocol published previously by our laboratory [20]. Isolated cells were placed in culture conditions in a 60 mm tissue culture dish (World Precision Instruments, Sarasota, FL) and kept in a humidified incubator (Heraeus Instruments, Newtown, CT) with 5% CO_2_ at 37°C. Cells were incubated with DMEM/F-12 (Invitrogen) supplemented with 10% FBS (Atlanta Biologicals, Lawrenceville, GA), 10 mM HEPES (Sigma, St. Louis, MO), 2 mM L-glutamine, 1 mM sodium pyruvate, 100 U/ml penicillin, and 100 μg/ml streptomycin. For all experiments the cells were maintained in primary culture for 3–7 days without passage. Prior to experimentation cells were rinsed with serum free media, serum starved overnight and kept in colorless serum free medium (Invitrogen, Carlsbad, CA) without antibiotics. Except for HEPES and FBS, all reagents were purchased from Invitrogen (Carlsbad, CA).

### COL-I coating on AFM probes

A 5 μm diameter glass microbead was glued to the tip of an atomic force microscope (AFM) probe (MLCT-O10, Santa Barbara, CA; Bruker Corp.) and the microbead tip coated with COL-I (Sigma, St. Louis, MO). The ECM coating protocol used was described by Lehenkari and Horton [21] and has been previously used in our laboratory [22–23]. Briefly, Polyethylene glycol (PEG, Sigma, St. Louis, MO) was used as a linker molecule between COL-I and the microbead. The probe was incubated with 10 mM PEG (5 min), washed with phosphate buffered saline (PBS), and then incubated with COL-I (1 mg/ml) for 5 min followed by rinsing with PBS. Each cantilever was calibrated after a given experiment using the thermal noise amplitude method of calibration [24–25]. The measured spring constants were between 10 and 20 pN/nm.

### Cell adhesion and E-modulus measurement with AFM

Monitoring of biomechanical and adhesive properties of VSMCs in real-time was performed using an Asylum AFM System (Model MFP-3D-BIO, Asylum Research, Santa Barbara, CA) that was mounted on an inverted microscope (Model IX81, Olympus America Inc.). All AFM measurements were conducted at room temperature (∼25°C). The AFM sampling parameters employed were 0.1 Hz sampling frequency, with an approach/retraction velocity of 320 nm/sec, 3200 nm traveling distance for one cycle of indentation and retraction, and approximately 1000–3000 pN loading force. Cells were randomly selected and indented at a site between the nucleus and cell margin to collect approximately 360 force curves over a 60 min experiment. To minimize drifting, after the probe was submerged in the cell bath, the AFM system was thermally and mechanically equilibrated for 60 min. After 60 min stabilization prior to AFM probe indentation, the AFM drift is approximately 1–2 microns in x, y, z directions during the one hour period needed for an experiment. In this study, the adhesion force and stiffness measurement was achieved by a continuous indentation protocol at 0.1 Hz sampling frequency in trigger mode. In trigger mode, we set the maximal indentation force exerted by AFM probe on cell surface at a preselected value, 1000 pN for instance, the AFM probe will then automatically retract as soon as the indentation force reaches the 1000 pN threshold during the indentation. This also protects cells from the potential damage caused by over indentation that could lead to cell membrane puncture. By setting this force threshold we offset the thermal drifting in the z direction. Meanwhile, the AFM probe used in this study was 5 μm diameter bead attached to the tip of the cantilever. This provides a contact area in excess of the 1–2 micron x-y drift and gives us a more spatially averaged measurement with less variability compared to a sharp tipped probe (30–50 nm tip dia). This helps to offset the drifting in x and y direction and will not significantly affect the accuracy of the cell elasticity measurement or detection of changes in adhesion. Each cell was subject to 10 min. of control measurements followed by 30 min. treatment with the vasoconstrictor angiotensin II (ANG-II; 10^−6^), the vasodilator adenosine (ADO; 10^−4^) or the nitric oxide (NO) donor (PANOate, 10^−6^ M).

### AFM force curve analysis

**Fig 1:**
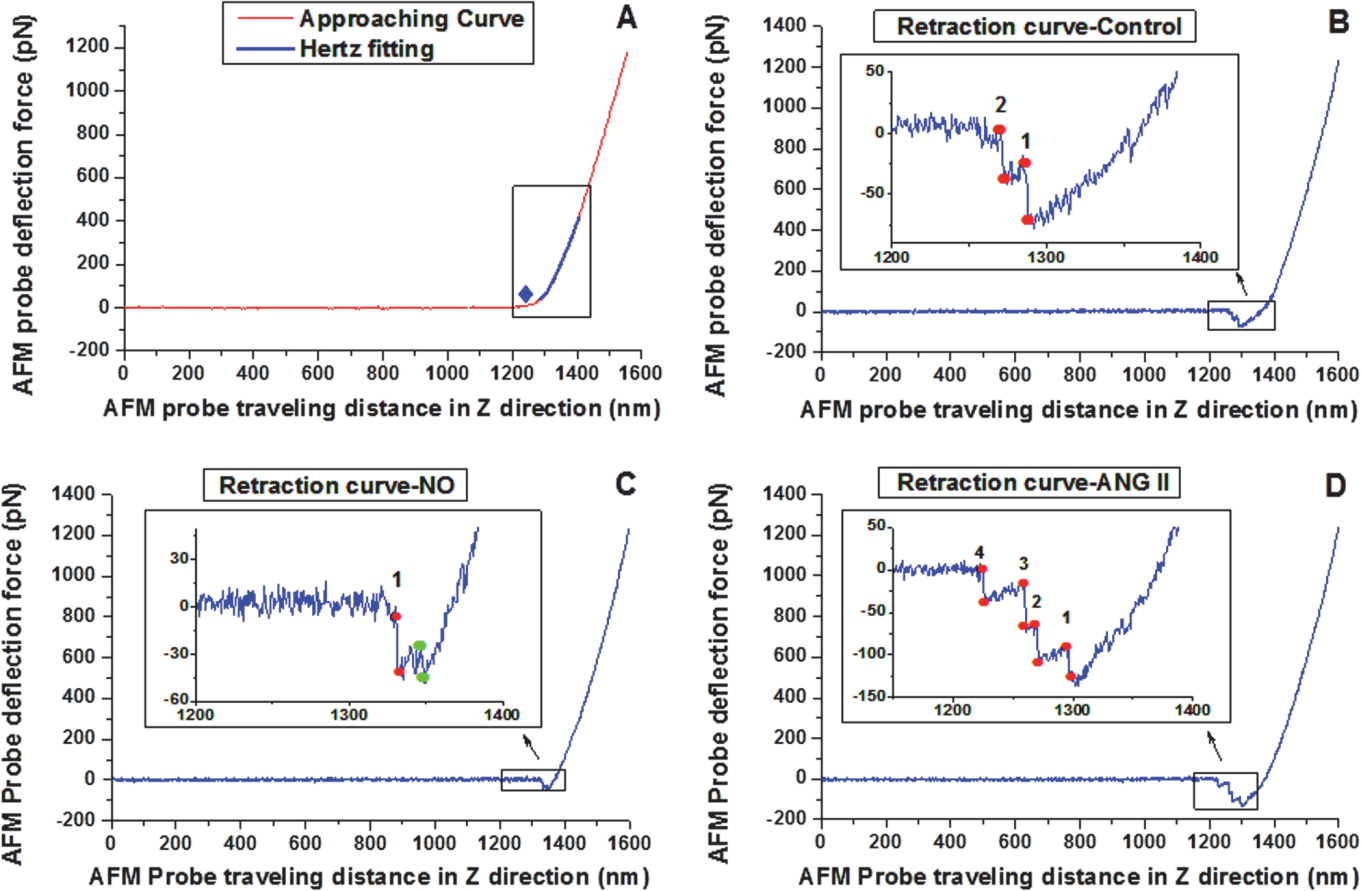
Representative force curve recorded by AFM. **(A)** An example of an approach curve recorded by AFM (red). The blue diamond in the black square is the estimated contact point with the VSMC where the AFM cantilever is in contact with the cell plasma membrane and begins to bend. The blue line is the Hertz fitting to the approach curve. **(B)** Representative retraction force curve recorded by AFM for pre-drug period (control). **(C)** Representative retraction force curves after treatment with NO. **(D)** Representative retraction force curve after treatment with ANG II. The height between the paired red spots was used to compute the adhesion force and the rupture number was used to evaluate the adhesion probability. The 1.5 fold of the average noise (signal fluctuation) was set as the threshold of adhesion force and only the rupture force higher than threshold was considered as the real unbinding force between cell membrane and AFM cantilever. The rupture indicated by two green spots was considered as noise and omitted in adhesion probability computation (**C)**. The snaps with low gap height were not the particular characteristics of the NO or ADO treated force curve, they also appeared in control and ANG II treated force curve and were omitted as well.

The analysis of force curves was automated using a proprietary software package written in Matlab (2014a, Mathworks). For estimating Young’s modulus (*E*-modulus) of the cell cortex, a length of the approach curve, following the initial point of contact, representing approximately 100–300 nm of AFM indentation into the VSMC, was fitted with a modified Hertz model (Eq. 1) as illustrated in **Fig. 1A** [26–27].

$$E=\frac{3(1-v^{2})}{4\sqrt{r_{b}}}\times \frac{F}{\delta^{\frac{3}{2}}}$$ (1)

Where *E* is the *E*-modulus, *F* is the force exerted by the AFM probe on the cell surface, *δ* is the indentation depth into the cell membrane, *r*_*b*_ is the radius of the spherical AFM tip, and *v* is the Poisson ratio for the cell. Cells were considered as a gel and the Poisson ratio *v* was assumed as 0.5 in this study [26]. Adhesion forces between COL-I and VSMC were determined as the product of cantilever spring constant and the height of ruptures (cantilever deflection) in retraction force curves (Hooke’s law) as illustrated in **Fig. 1B-1D**. Only the ruptures with a threshold height greater than 1.5 times of average noise fluctuation within the AFM retraction curve (normally around 18 pN) were analyzed and regarded as specific adhesions between the membrane and AFM probe. The adhesion probability was computed as the number of effective adhesion events per AFM retraction curve.

### Statistical Analysis

Data are reported as the mean ± SEM. Statistically significant differences in the elasticity or adhesion probability between the pre-drug controls and post-drug treatments were analyzed with one way ANOVA. A value of *P<0.05* was considered significant.

## Results

### VSMC E-modulus and adhesion probability increases in response to the vasoconstrictor ANG II

**Fig 2:**
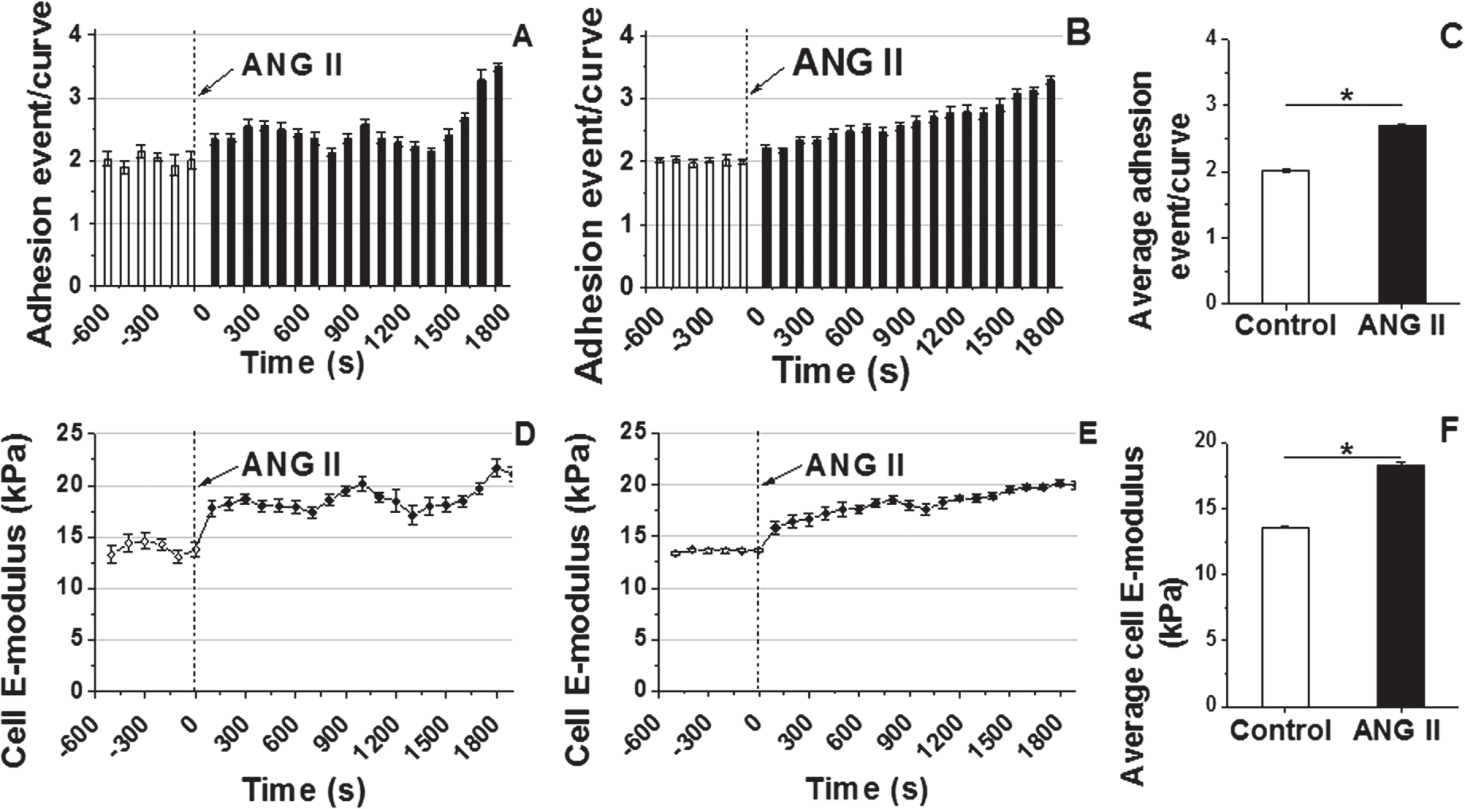
Continuous real-time recordings of E-modulus and adhesion probability of VSMC to COL-I coated AFM probe following stimulation with ANG II. The adhesion probability is presented as the number of adhesion events per AFM retraction curve. **(A)** A representative individual example shows the increase in VSMC adhesion probability after stimulation with ANG II. **(B)** Average adhesion probability before and after addition of ANG II for experimental group (n = 10). **(C)** Average adhesion probability significantly increased after ANG II treatment. Data were summed over 1800 s and were presented as mean ± SEM (n = 10, *P<0.05). **(D)** A representative single cell record of VSMC E-modulus shows the immediate increase in cell E-modulus after the addition of ANG II in cell bath (10^−6^ M). **(E)** Alteration in group average of VSMC E-modulus before and after stimulation with ANG II (n = 10). **(F)** Average E-modulus summed across all time points for the group significantly increased after addition of ANG II. Data were summed over 1800 s and were presented as mean ± SEM (n = 10, *P<0.05.

**Fig. 2** shows the alteration in E-modulus and adhesion probability of VSMC to the COL-I coated AFM probe upon ANG II stimulation. Following addition of ANG II to the cell bath the adhesion probability (i.e. number of adhesion events per AFM retraction curve) to COL-I progressively increased over a 30 min period as shown in a representative example of an individual experiment (**Fig. 2A**) and for the entire group of cells (n = 10) tested (**Fig. 2B, 2C**). During the 10 min pre-drug control period the cell adhesion probability was approximately 2 events per curve and increased to approximately 3.1 events per curve 30 min post-exposure to ANG II stimulation. The averaged adhesion probability summed over the entire period of ANG II exposure was significantly increased by ∼33% (**Fig. 2C**, n = 10, *P<0.05). To confirm the effect of ANG II on VSMC E-modulus was occurring in combination with the ANG II induced increase in adhesion probability, cell E-modulus was estimated by fitting a modified Hertz model to the AFM approaching force curve. As shown in a representative example of an individual experiment (**Fig. 2D**), exposure to ANG II rapidly increased cell E-modulus from ∼13.5 kPa to ∼18.5 kPa within 2 min and further increased to ∼22 kPa by 30 min. The group average of E-modulus exhibited an increasing trend following exposure to ANG II (**Fig. 2E**, n = 10). **Fig. 2F** shows the increase (∼35%) in VSMC E-modulus averaged for the entire group of experiments (n = 10, *P<0.05).

### No alteration in VSMC E-modulus or adhesion probability in sham control experiment

**Fig 3:**
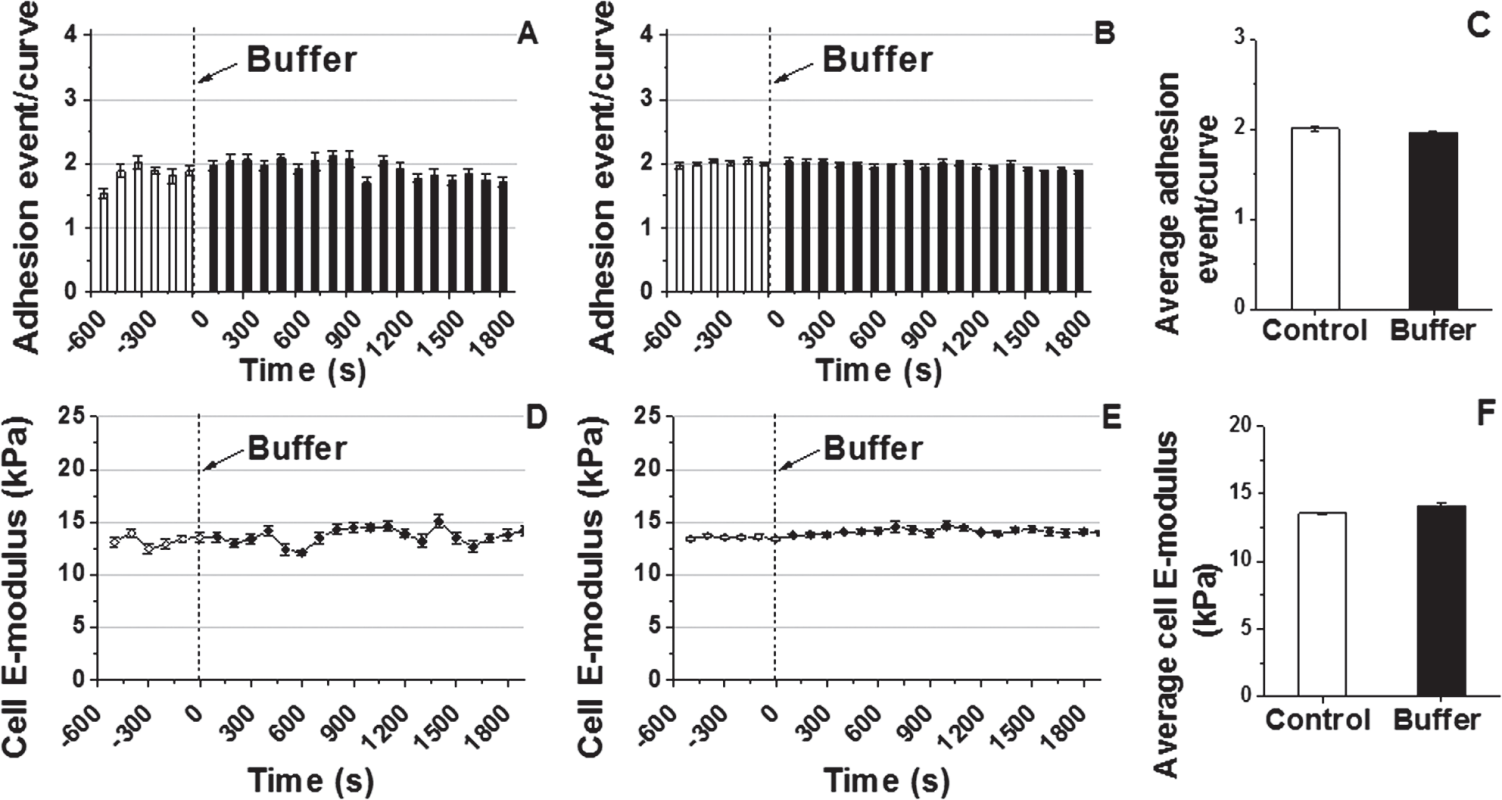
Continuous real-time recordings of E-modulus and adhesion probability of VSMC to COL-I coated AFM probe in sham control experiments. **(A)** A representative individual example shows no effect on adhesion probability after addition of vehicle buffer. **(B)** No change in adhesion probability was shown in the group average adhesion probability before and after addition of vehicle buffer (n = 10). **(C)** Average adhesion probability summed across all time points for the group of VSMCs before and after addition of vehicle buffer (n = 10, *P>0.05*). **(D)** A representative single cell measurement shows no changes in E-modulus before and after addition of vehicle buffer in cell bath. **(E)** Group average VSMC E-modulus did not change before and after addition of vehicle buffer in cell bath (n = 10). **(F)** No significant difference was shown in the average E-modulus summed for all time points before and after addition of vehicle buffer (n = 10, *P>0.05*). Data were presented as mean ± SEM.

To test if the cell adhesion or E-modulus were altered during the course of a repetitive indentation experiment, a sham control study was conducted with the addition of vehicle buffer to the cell bath. **Fig. 3A** shows continuous real-time recordings of adhesion probability of VSMC to a COL-I coated AFM probe in a representative sham control experiment. No significant alterations were exhibited during the entire time course of the experiment. The group average adhesion probability (**Fig. 3B**) and the average adhesion probability summed across all time points before and after addition of vehicle buffer showed no significant alterations (**Fig. 3C**, n = 10, *P>0.05*). The representative continuous real time measurement (**Fig. 3D**) and the group average (**Fig. 3E**) show the VSMCs’ E-modulus was approximately ∼12 kPa with no significant alteration throughout the sham control experimental duration. **Fig. 3F** summarizes E-modulus across all time points before and after addition of vehicle buffer to the cell bath (n = 10, *P>0.05*).

### VSMC adhesion probability decreases in response to vasodilation by ADO and NO

**Fig 4:**
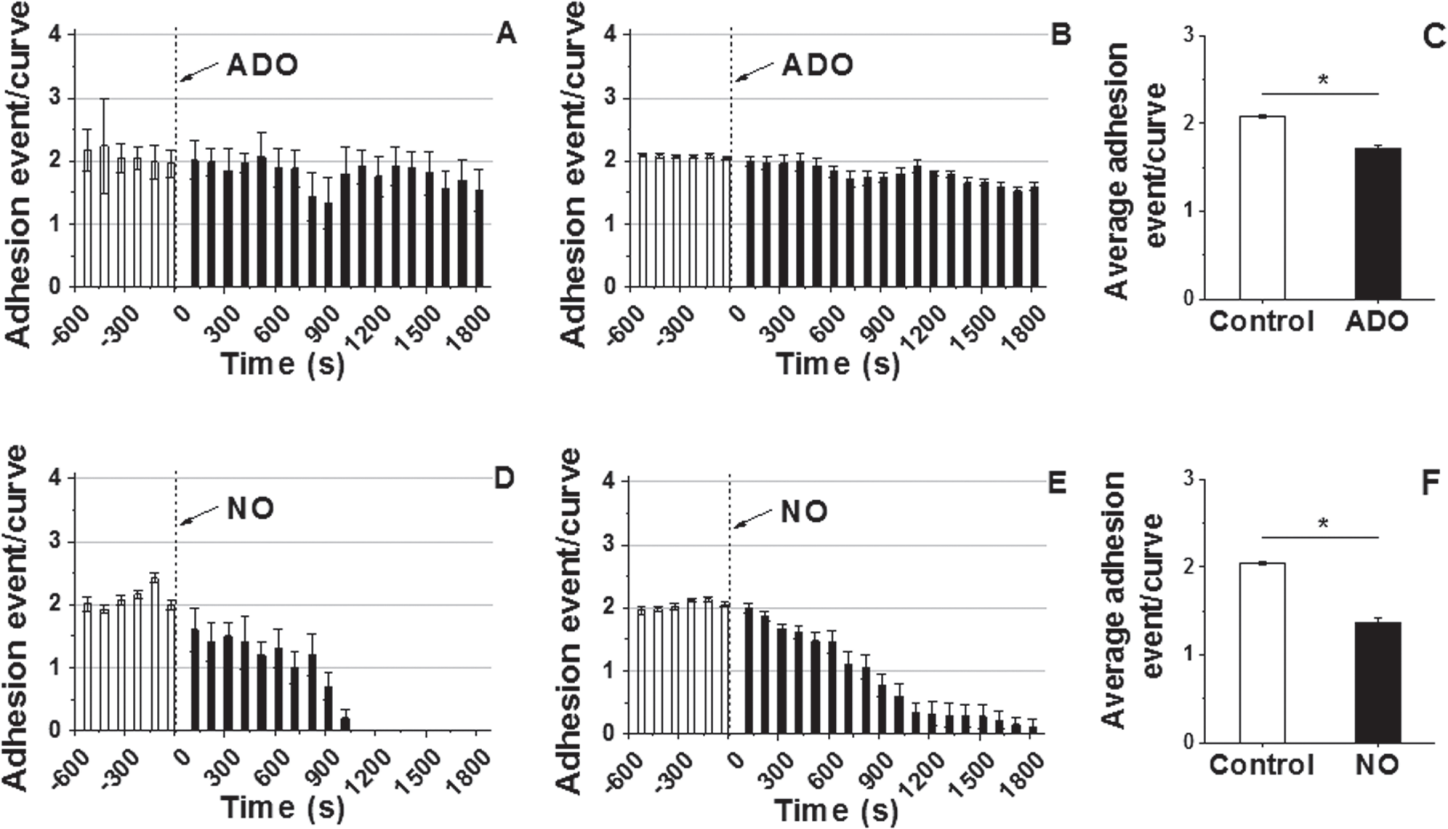
Continuous real-time recordings of adhesion probability of VSMC to COL-I coated AFM probe following ADO or NO administration. **(A)** A representative individual experiment shows the decrease in the adhesion probability after introduction of ADO in the cell bath. **(B)** Decrease in the group average adhesion probability after addition of ADO in the cell bath (n = 10). **(C)** Average adhesion probability showed a significant decrease in adhesion probability following addition of ADO. Data were summed over 1800 s and were presented as mean ± SEM (n = 10, *P<0.05). **(D)** NO donor dramatically reduced adhesion probability as shown in a representative individual example. **(E)** Average adhesion events decreased after treatment with NO (n = 10). **(F)** Average adhesion probability shows a significant decrease in adhesion probability after the addition of NO donor to the cell bath. Data were summed over 1800 s and were presented as mean ± SEM (n = 10, *P<0.05).

To determine if pharmacological vasodilation exerts opposite effects on VSMC adhesion behavior compared to ANG II, two potent vasodilators, ADO or NO were used to relax the VSMC during the continuous AFM indentation cycles. Results showed that the adhesion probability of VSMC to COL-I significantly decreased following addition of ADO to the cell bath from approximately 2 events per curve during the control period to 1.5 events per curve at 30 min post drug period as shown in a representative individual experiment (**Fig. 4A**). The group average data confirmed the decrease in adhesion probability upon treatment with ADO (**Fig. 4B**, n = 10). Average adhesion probability summed across all time points shows the reduction (−17%) in adhesion probability of VSMC to COL-I following ADO treatment (**Fig. 4C** n = 10, *P<0.05). The effect of the NO donor (PANOate) on the adhesion probability between VSMC and COL-I is shown in a representative example (**Fig. 4D**). The adhesion probability decreased rapidly after addition of NO donor to the cell bath. After 15 min stimulation with the NO donor adhesion was decreased to such an extent that significant adhesions were no longer detectable between VSMC and COL-I. The group average data showed the reduction trend (**Fig. 2E**, n = 10) and a significant decrease (∼33%) in the adhesion probability after stimulation with NO (**Fig. 2F**, n = 10, *P<0.05).

### VSMC E-modulus decreases in response to vasodilation with ADO and NO

**Fig 5:**
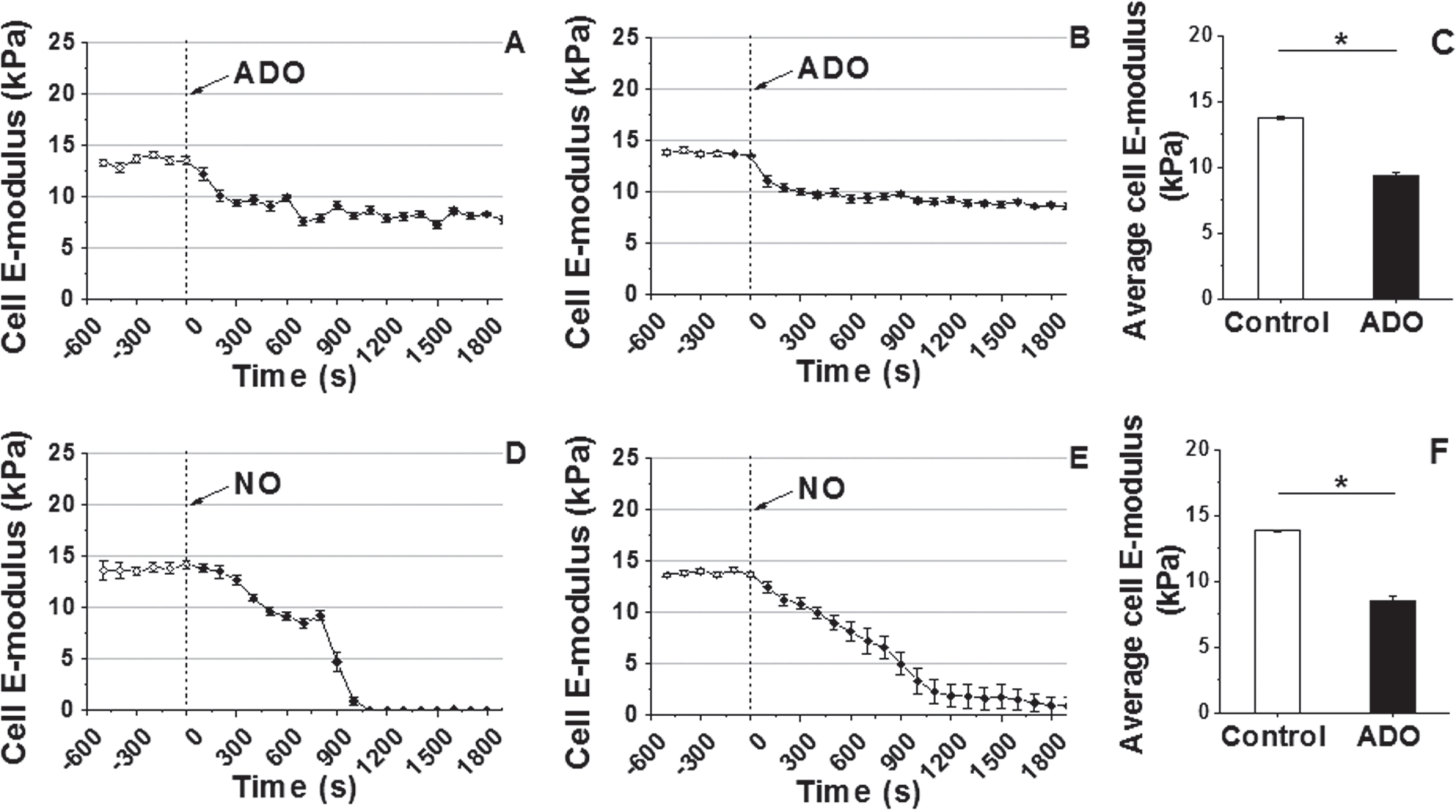
Continuous real-time VSMC E-modulus recordings following ADO or NO. **(A)** VSMC E-modulus immediately reduced after addition of ADO (10^−4^ M) as shown in a representative example. **(B)** Group average of VSMC E-modulus after addition of ADO (n = 10) in cell bath. **(C)** Average E-modulus summed across all time points for the group of VSMCs before and after ADO (n = 10, *P<0.05). **(D)** A representative single cell measurement shows decrease in VSMC E-modulus following addition of NO to the cell bath. **(E)** Alteration in group average of VSMC E-modulus before and after addition of NO (n = 10). **(F)** Average E-modulus summed for all time points before and after addition of NO showed a significant decrease in VSMC E-modulus (n = 10, *P<0.05). Data were presented as mean ± SEM.

The effects of the vasodilators ADO or NO on VSMC E-modulus are shown in **Fig. 5**. A representative example of a single cell record of VSMC E-modulus before and after addition of ADO showed that the exposure to ADO lead to a significant decrease in cell E-modulus from ∼13 kPa to ∼8 kPa (**Fig. 5A)**. The group average of VSMC E-modulus confirmed the decrease in cell E-modulus upon exposure to ADO (**Fig. 5B**, n = 10). Average E-modulus summed across all time points for the group of VSMCs before and after addition of ADO also demonstrated a significant decrease by −31% (**Fig. 5C**, n = 10, *P<0.05). Compared to ADO, the NO donor had a more pronounced effect on cell E-modulus, in a representative experimental example, NO reduced cell stiffness to barely detectable levels indicative of a loss of cortical stiffness (**Fig. 5D**). The group average of VSMC E-modulus alteration before and after addition of NO in the cell bath confirmed the decreasing trend in cell E-modulus following NO treatment (**Fig. 5E**, n = 10). **Fig. 5F** presents the significant decrease (−38%) in the average E-modulus summed over the entire period of NO exposure (n = 10, *P<0.05).

## Discussion

VSMCs within the walls of arteries sense, transduce, and generate mechanical forces. They detect both local and soluble vasoactive signals and respond by alterations in vascular smooth muscle tone that is fundamental to regulation of vessel diameter. Integrin-mediated cell adhesion to ECM at focal adhesion sites has been identified as one major mechanosensory site as well as an important region for transmission of mechanical forces and cell signals [20,23]. Of relevance to our hypothesis, the integrins maintain the integrity of cell adhesion and act as logical sites for transmission of force from the external environment to the cytoskeleton and for transmission of generated cellular contractile force to ECM. In our previous studies, we demonstrated that adhesion between VSMCs and the ECM protein FN was regulated by vasoactive agonists [4,28]. The vasoconstrictor ANG II up-regulated cell adhesion to FN and induced a significant increase in cell E-modulus. In addition, ANG II increased the density and orientation of actin stress fibers in the VSMC cell cortex. In contrast, the vasodilator ADO down-regulated cell adhesion, cell E-modulus, and reduced the density and orientation of the actin stress fibers in the cell cortex. Our findings in the present study are consistent with these observations as applied to COL-I and further support the concept that ECM adhesion in VSMC is regulated in tandem with changes in VSMC contractile activation.

The current study employed AFM for real time monitoring of cell E-modulus and adhesion similar to our prior VSMC-FN study [4]. Cellular E-modulus was evaluated by AFM with a well established method [29]. 100–300 nm depth of cell cortex was indented with 5μm spherical AFM probe and a modified Hertz model was fitted to the approaching force curve since cell cortex is normally around 300 nm thickness [30]. Our results clearly indicate that ANG II significantly increases both VSMC adhesion probability to COL-I and VSMC E-modulus, whereas ADO and NO decrease adhesion to COL-I and E-modulus of VSMCs. Our results suggest the integrin-mediated interaction between the ECM and VSMC is modulated by G-protein coupled receptor signaling that is also linked to activation of the contractile machinery of the cell. Thus, there appears to be a receptor dependent inside-out pathway for modulation of adhesion during VSMC activation.

The alterations in VSMC adhesion to COL-I and FN [4] following treatment with vasoactive agents (constrictor and dilator) may function as a means of tuning adhesion to cellular force generation. As such, when the VSMC is generating force, the focal adhesion sites adhere more tightly and become more efficient sites for force transmission. Likewise when force generation declines this is accompanied by decreased adhesion. In addition, this increase in adhesion would be anticipated to augment outside-in signaling through VSMC focal adhesions [31–33]. The changes in cell E-modulus following treatment with ANG II, ADO or NO are consistent with rapid remodeling in the cortical actin cytoskeleton as we have previously shown [28]. This compartment of actin is generally considered as non-contractile, and has been demonstrated to rapidly remodel during activation of smooth muscle contraction and is required for full development of a VSMC contractile event. It is noteworthy that the effects of adenosine were less pronounced than those of NO. This is consistent with NO being a more potent vasodilator than adenosine. In addition, NO may act more rapidly because it does not act through a G-protein coupled receptor, as does adenosine. The ability of NO to directly activate cyclic GMP as opposed to receptor-mediated activation of cyclic AMP by adenosine could also accounts for differences in rate and magnitude over the time course studied.

Consistent with our findings in VSMC, Gunst et al. have previously reported that dynamic cytoskeletal changes occur during contraction of pulmonary smooth muscle cells. They report that a non-contractile actin compartment of the cytoskeleton was actively involved in determining development of contractile tone and those changes in actin binding proteins and focal adhesion proteins are an orchestrated component of airway smooth muscle cell contraction [34–36]. Similarly, Morgan et al. have also reported that actin cytoskeleton remodeling [37] and reorganization occurs within cell cortex and the focal adhesion complex during contraction of VSMC [38–39]. More recently, a similar reorganization in cytoskeletal structure during myogenic constriction of cerebral arteries was reported [40]. Collectively, these findings support the general concept that cell contraction in smooth muscle is strongly coupled to changes in cell adhesion, focal adhesion proteins and cell stiffness due to cortical cytoskeletal remodeling of the submembranous actin compartment.

In our study we used isolated VSMCs and maintained them in primary culture without passage to minimize any phenotypic changes. The use of our AFM protocol required that the VSMCs firmly attach to the substrate and the short-term primary culture allows for this period of attachment as well as recovery from the enzymatic effects of the isolation process. Ultimately, measurement of cell adhesion and E-modulus of VSMCs in their native environment would be ideal but with the AFM this is still technically not achievable.

In summary, our results showed that the vasoconstrictor ANG II increased both VSMC E-modulus and the probability of cell adhesion to COL-I. In contrast, the vasodilators ADO or the NO donor PANOate decreased VSMC E-modulus and reduced COL-I adhesion probability. These observations support our hypothesis that integrin-mediated VSMC adhesion to the ECM protein COL-I is dynamically regulated in parallel with the VSMC contractile activation. This supports a strong association between the signal transduction pathways involved in cell contraction and cell adhesion. Obviously, additional work is required to identify the molecular mechanisms underlying the coupling of VSMC adhesion and E-modulus, and to determine whether these pathways are parallel or share similar intermediate steps. It is of interest that in aging and hypertension, both cell stiffness and adhesion of VSMC from the aorta are significantly enhanced [18–19]. Thus, identifying these mechanisms may help to provide valuable clues in cardiovascular disease relating to the cause of vascular stiffness, thereby, assisting in developing novel treatment strategies.
